# Hermite-Hadamard type inequalities for the generalized k-fractional integral operators

**DOI:** 10.1186/s13660-017-1476-y

**Published:** 2017-09-04

**Authors:** Erhan Set, Junesang Choi, Abdurrahman Gözpinar

**Affiliations:** 1 Department of Mathematics, Faculty of Science and Arts, Ordu University, Ordu, Turkey; 2 Department of Mathematics, Dongguk University, Gyeongju, 38066 Republic of Korea

**Keywords:** 26A33, 26D10, 26D15, 33B20, Gamma function, k-gamma function, convex function, Hermite-Hadamard type inequalities, Riemann-Liouville fractional integrals, generalized k-fractional integral operators

## Abstract

We firstly give a modification of the known Hermite-Hadamard type inequalities for the generalized k-fractional integral operators of a function with respect to another function. We secondly establish several Hermite-Hadamard type inequalities for the generalized k-fractional integral operators of a function with respect to another function. The results presented here, being very general, are pointed out to be specialized to yield some known results. Relevant connections of the various results presented here with those involving relatively simple fractional integral operators are also indicated.

## Introduction and preliminaries

A function $f:I \rightarrow \mathbb{R}$ is said to be convex if the following inequality holds:

1 $$ f \bigl( tx+ ( 1-t ) y \bigr) \leq tf ( x ) + ( 1-t ) f ( y ) \quad \bigl(x, y\in I; t\in [ 0,1 ] \bigr), $$

where *I* is an interval in the real line $\mathbb{R}$. Here and in the following, let $\mathbb{C}$, $\mathbb{R}$, $\mathbb{R}^{+}$, and $\mathbb{N}$ be the sets of complex numbers, real numbers, positive real numbers, and positive integers, and let $\mathbb{N}_{0}:=\mathbb{N} \cup \{0\}$ and $\mathbb{R}^{+}_{0}:= \mathbb{R}^{+} \cup \{0\}$.

One of the best-known inequalities for convex functions is the following Hermite-Hadamard inequality: If $f:I\subseteq \mathbb{R}\rightarrow \mathbb{R}$ (*I* is an interval) is a convex function and $a, b \in I$ with $a< b$, then

2 $$\begin{aligned} f \biggl( \frac{a+b}{2} \biggr) \leq \frac{1}{b-a} \int_{a}^{b}f ( x )\,dx\leq \frac{f ( a ) +f ( b ) }{2}. \end{aligned}$$

The Hermite-Hadamard inequality in (2) has attracted many mathematicians’ attention who have presented a variety of generalizations, extensions, and variants, which are called Hermite-Hadamard type inequalities (see, *e.g.*, [1–4] and the references cited therein).

Recently, several Hermite-Hadamard type inequalities associated with fractional integrals have been investigated. Here, we aim to establish several generalized Hermite-Hadamard type integral inequalities for the generalized k-fractional integral operators with respect to another function. The results presented here, being very general, are also pointed out to be specialized to yield some known results. Relevant connections of the various results presented here with those involving relatively simple fractional integral operators are also indicated.

To do this, we recall some definitions and known results. Let $[a,b]$ ($-\infty < a< b<\infty $) be a finite interval on the real axis $\mathbb{R}$. The Riemann-Liouville fractional integrals $J_{a+}^{ \alpha }f$ and $J_{b-}^{\alpha }f$ of order $\alpha \in \mathbb{C}$ ($\Re (\alpha )>0$) with $a\geq 0$ and $b >0$ are defined, respectively, by

3 $$ \bigl( J_{a+}^{\alpha }f \bigr) (x):= \frac{1}{\Gamma (\alpha )} \int_{a} ^{x} ( x-t ) ^{\alpha -1} f(t)\,dt \quad \bigl(x>a; \Re ( \alpha )>0 \bigr) $$

and

4 $$ \bigl( J_{b-}^{\alpha }f \bigr) (x):= \frac{1}{\Gamma (\alpha )} \int_{x} ^{b} ( t-x ) ^{\alpha -1}f(t)\,dt \quad \bigl(x< b; \Re (\alpha )>0 \bigr). $$

Here $\Gamma (\alpha )$ is the familiar Gamma function (see, *e.g.*, [5], Section 1.1). For more details and properties of the fractional integral operators (3) and (4), we refer the reader, for example, to [6–14] and the references therein.

Let $\Omega =[a,b]$ ($-\infty \leq a< b \leq \infty $) be a finite or infinite interval on the real axis $\mathbb{R}$. We denote by $L_{p}(a,b)$ ($1\leq p \leq \infty $) the set of those Lebesgue complex-valued measurable functions *f* on Ω for which $\Vert f\Vert _{p} <\infty $, where

5 $$ \Vert f\Vert _{p} = \biggl( \int_{a}^{b} \bigl\vert f(t) \bigr\vert ^{p}\,dt \biggr) ^{1/p} \quad (1 \leq p < \infty ) $$

and

6 $$ \Vert f\Vert _{\infty }= \operatorname{ess} \sup _{a \leq x \leq b} \bigl\vert f(x) \bigr\vert . $$

In particular, $L_{1}(a,b):=L(a,b)$.

Raina [15] introduced a class of functions defined formally by

7 $$ \mathcal{F}_{\rho ,\lambda }^{\sigma }(x) = \mathcal{F}_{\rho ,\lambda }^{\sigma (0),\sigma (1),\ldots } (x)= \sum_{m=0}^{\infty } \frac{ \sigma (m)}{\Gamma (\rho m+\lambda )}x^{m} \quad \bigl( \rho , \lambda \in \mathbb{R}^{+}; x \in \mathbb{R} \bigr) , $$

where the coefficients $\sigma (m)\in \mathbb{R}^{+} $ ($m\in \mathbb{N}_{0} $) form a bounded sequence. With the help of (7), Raina [15] and Agarwal *et al.* [16] defined, respectively, the following left-sided and right-sided fractional integral operators:

8 $$ \bigl( \mathcal{J}_{\rho ,\lambda ,a+;w}^{\sigma }\varphi \bigr) (x)= \int_{a}^{x}(x-t)^{\lambda -1} \mathcal{F}_{\rho ,\lambda }^{\sigma } \bigl[w(x-t)^{\rho } \bigr] \varphi (t)\,dt \quad (x>a>0) $$

and

9 $$ \bigl( \mathcal{J}_{\rho ,\lambda ,b-;w}^{\sigma }\varphi \bigr) (x)= \int_{x}^{b}(t-x)^{\lambda -1} \mathcal{F}_{\rho ,\lambda }^{\sigma } \bigl[w(t-x)^{\rho } \bigr] \varphi (t)\,dt \quad (0< x< b), $$

where $\lambda , \rho \in \mathbb{R}^{+}$, $w\in \mathbb{R}$, and $\varphi (t)$ is a function such that the integrals on the right sides exist. Recently, certain new and interesting inequalities involving these fractional operators have appeared in the literature (see, *e.g.*, [16–20]).

It is easy to verify that $\mathcal{J}_{\rho ,\lambda ,a+;w}^{\sigma }\varphi (x)$ and $\mathcal{J}_{\rho ,\lambda ,b-;w}^{\sigma }\varphi (x)$ are bounded integral operators on $L(a,b)$, if

10 $$ \mathfrak{M}:=\mathcal{F}_{\rho ,\lambda +1}^{\sigma } \bigl[w(b-a)^{ \rho } \bigr] < \infty . $$

In fact,

11 $$ \bigl\Vert \mathcal{J}_{\rho ,\lambda ,a+;w}^{\sigma }\varphi (x) \bigr\Vert _{1} \leq \mathfrak{M} (b-a)^{\lambda }\Vert \varphi \Vert _{1} \quad \bigl(\varphi \in L(a,b) \bigr) $$

and

12 $$ \bigl\Vert \mathcal{J}_{\rho ,\lambda ,b-;w}^{\sigma }\varphi (x) \bigr\Vert _{1} \leq \mathfrak{M} (b-a)^{\lambda }\Vert \varphi \Vert _{1} \quad \bigl(\varphi \in L(a,b) \bigr). $$

Here, many useful fractional integral operators can be obtained by specializing the function $\mathcal{F}_{\rho ,\lambda }^{\sigma }(x)$. For instance, the classical Riemann-Liouville fractional integrals $J_{a+}^{\alpha }$ and $J_{b-}^{\alpha }$ of order *α* follow easily by setting $\lambda =\alpha $, $\sigma (0)=1$, and $w=0$ in (8) and (9).

Budak *et al.* [21] established a new identity involving the fractional integral operators (8) and (9) asserted by the following lemma.

### Lemma 1

*Let* $f :[a,b]\to \mathbb{R}$ *be a differentiable mapping on* $(a,b)$ *with* $a< b$ *and* $f{'} \in L(a,b)$. *Then*

13 $$ \begin{aligned}[b] &\frac{2^{\lambda -1}}{(b-a)^{\lambda }\mathcal{F}_{\rho ,\lambda +1} ^{\sigma } [w(\frac{b-a}{2})^{\rho }]} \bigl[ \mathcal{J}^{\sigma } _{\rho ,\lambda ,{\frac{a+b}{2}}-;w}f(a)+\mathcal{J}^{\sigma }_{ \rho ,\lambda ,{\frac{a+b}{2}}+;w }f(b) \bigr] -f \biggl( \frac{a+b}{2} \biggr) \\ & \quad = \frac{(b-a)}{4\mathcal{F}_{\rho ,\lambda +1}^{\sigma } [w( \frac{b-a}{2})^{\rho }]} \biggl\{ \int_{0}^{1}t^{\lambda }\mathcal{F} _{\rho ,\lambda +1}^{\sigma } \biggl[w \biggl(\frac{b-a}{2} \biggr)^{\rho }t^{\rho } \biggr] f {'} \biggl( \frac{t}{2}a+\frac{2-t}{2}b \biggr)\,dt \\ & \qquad {}- \int_{0}^{1}t^{\lambda }\mathcal{F}_{\rho ,\lambda +1} ^{\sigma } \biggl[ w \biggl(\frac{b-a}{2} \biggr)^{\rho }t^{\rho } \biggr] f{'} \biggl( \frac{2-t}{2}a+ \frac{t}{2}b \biggr)\,dt \biggr\} . \end{aligned} $$

We recall the following generalized fractional integral operators (see, *e.g.*, [12], Section 18.2). Let $g:[a,b]\longrightarrow \mathbb{R}$ ($-\infty \leq a< b \leq \infty $) be an increasing and positive function having a continuous derivative $g'$ on $(a,b)$. The left- and right-sided generalized fractional integrals of *f* with respect to the function *g* on $[a,b]$ of order *α* are defined, respectively, by

14 $$\begin{aligned}& \bigl( \mathcal{I}_{a+;g}^{\alpha }f \bigr) (x)= \frac{1}{\Gamma ( \alpha )} \int_{a}^{x} \frac{g'(\tau )f(\tau )}{ ( g(x)-g(\tau ) ) ^{1-\alpha }}\,d\tau \quad \bigl( \Re (\alpha )>0; x>a \bigr) \end{aligned}$$

and

15 $$\begin{aligned}& \bigl( \mathcal{I}_{b-;g}^{\alpha }f \bigr) (x)= \frac{1}{\Gamma ( \alpha )} \int_{x}^{b} \frac{g'(\tau )f(\tau )}{ ( g(\tau )-g(x) ) ^{1-\alpha }}\,d\tau \quad \bigl(\Re (\alpha )>0; x< b \bigr), \end{aligned}$$

provided that the integrals exist. The integrals (14)) and (15) are usually called fractional integrals of a function *f* by a function *g* of the order *α*. Choosing $g(x)=x$ in (14) and (15) reduces to the Riemann-Liouville fractional integrals (3) and (4), respectively. Setting $g(x)=\ln x$ in (14) and (15) reduces to yield Hadamard fractional integrals (see, *e.g.*, [12], pp.329-330). Jleli and Samet [22] presented ceratin Hermite-Hadamard type inequalities for the integrals (14)) and (15).

Díaz and Pariguan [23] introduced and investigated the so-called k-gamma function

16 $$ \Gamma_{\mathtt{k}}(x):= \int_{0}^{\infty }t^{x-1}e^{-\frac{t^{ \mathtt{k}}}{\mathtt{k}}}\, dt \quad \bigl( \Re (x)>0;\mathtt{k} \in \mathbb{R}^{+} \bigr) . $$

We recall some properties for the k-gamma function:

17 $$\begin{aligned}& \Gamma_{\mathtt{k}}(x)=\mathtt{k}^{\frac{x}{\mathtt{k}}-1}\Gamma \biggl( \frac{x}{\mathtt{k}} \biggr) ; \end{aligned}$$

18 $$\begin{aligned}& \Gamma_{\mathtt{k}}(\mathtt{k})=1 \quad \text{and} \quad \Gamma_{\mathtt{k}}(x+\mathtt{k})=x\Gamma_{\mathtt{k}}(x). \end{aligned}$$

Using the k-gamma function, Tunç *et al.* [24] introduced a class of functions defined by

19 $$ \mathcal{F}_{\rho ,\lambda }^{\sigma ,\mathtt{k}}(x) = \sum _{m=0}^{ \infty } \frac{\sigma (m)}{\mathtt{k} \Gamma_{\mathtt{k}}(\rho \mathtt{k} m+\lambda )}x^{m} \quad \bigl( \mathtt{k},\rho , \lambda \in \mathbb{R}^{+}; \vert x\vert < \infty \bigr) , $$

where $\sigma (m) \in \mathbb{R}^{+}$ ($m\in \mathbb{N}_{0}$) is a bounded sequence as given in (7). Tunç *et al.* [24] used the function (19) to define the left-sided and right-sided generalized k-fractional integral operators with respect to another function as follows: Let $\mathtt{k},\rho ,\lambda \in \mathbb{R}^{+}$ and $w\in \mathbb{R}$. Also, let $g:[a,b]\rightarrow \mathbb{R}$ be an increasing and positive function having a continuous derivative $g'$ on $(a,b)$. Then the left- and right-sided generalized k-fractional integrals of *f* with respect to the function *g* on $[a,b]$ are defined, respectively, by

20 $$\begin{aligned}& \mathcal{J}_{\rho ,\lambda ,a+;w}^{\sigma ,\mathtt{k},g}f(x)= \int _{a}^{x} \frac{g{'}(t)}{ ( g(x)-g(t) ) ^{1-\frac{\lambda }{ \mathtt{k}}}} \mathcal{F}_{\rho ,\lambda }^{\sigma ,\mathtt{k}} \bigl[w \bigl(g(x)-g(t) \bigr)^{ \rho } \bigr] f(t)\, dt \quad (x>a) \end{aligned}$$

and

21 $$\begin{aligned}& \mathcal{J}_{\rho ,\lambda ,b-;w}^{\sigma ,\mathtt{k},g}f(x)= \int _{x}^{b} \frac{g{'}(t)}{ ( g(t)-g(x) ) ^{1-\frac{\lambda }{ \mathtt{k}}}} \mathcal{F}_{\rho ,\lambda }^{\sigma ,\mathtt{k}} \bigl[w \bigl(g(t)-g(x) \bigr)^{ \rho } \bigr] f(t)\,dt \quad (x< b). \end{aligned}$$

Setting $\mathtt{k}=1$, $g(t)=t$, $g(t)=\ln t$, and $g(t)= \frac{t^{s+1}}{s+1}$ $(s\in \mathbb{R}\setminus \{-1\})$ in the integral operator (20) gives the generalized fractional integral operator of *f* with respect to the function *g*, the generalized k-fractional integral operator of *f* on $[a,b]$, the generalized Hadamard k-fractional integral operator of *f*, and the generalized $(\mathtt{k},s)$-fractional integral operator of *f* on $[a,b]$, respectively, as follows:

22 $$\begin{aligned}& \mathcal{J}_{\rho ,\lambda ,a+;w}^{\sigma ,g}f(x)= \int_{a}^{x} \frac{g {'}(t)}{ ( g(x)-g(t) ) ^{1-\lambda }} \mathcal{F}_{\rho , \lambda }^{\sigma } \bigl[w \bigl(g(x)-g(t) \bigr)^{\rho } \bigr] f(t)\,dt \quad (x>a); \end{aligned}$$

23 $$\begin{aligned}& \mathcal{J}_{\rho ,\lambda ,a+;w}^{\sigma ,\mathtt{k}}f(x)= \int_{a} ^{x} (x-t)^{\frac{\lambda }{\mathtt{k}}-1} \mathcal{F}_{\rho ,\lambda }^{\sigma ,\mathtt{k}} \bigl[w(x-t)^{\rho } \bigr] f(t) \,dt \quad (x>a); \end{aligned}$$

24 $$\begin{aligned}& \mathcal{H}_{\rho ,\lambda ,a+;w}^{\sigma ,\mathtt{k}}f(x)= \int_{a} ^{x} \biggl( \ln \frac{x}{t} \biggr) ^{\frac{\lambda }{\mathtt{k}}-1} \mathcal{F}_{\rho ,\lambda }^{\sigma ,\mathtt{k}} \biggl[ w \biggl( \ln \frac{x}{t} \biggr) ^{\rho } \biggr] f(t)\,\frac{dt}{t} \quad (x>a); \end{aligned}$$

25 $$\begin{aligned}& \begin{aligned}[b] {}^{s} \! \mathcal{J}_{\rho ,\lambda ,a+;w}^{\sigma ,\mathtt{k}}f(x)={}&(s+1)^{1-\frac{ \lambda }{\mathtt{k}}} \\ & {} \times \int_{a}^{x} \bigl(x^{s+1}-t^{s+1} \bigr)^{\frac{\lambda }{ \mathtt{k}}-1} t^{r}\mathcal{F}_{\rho ,\lambda }^{\sigma ,\mathtt{k}} \biggl[ w \biggl( \frac{x^{s+1}-t^{s+1}}{s+1} \biggr) ^{\rho } \biggr] f(t)\, dt \quad (x>a). \end{aligned} \end{aligned}$$

The special cases of (20) and (21) when $\mathtt{k}=1$ and $g(t)=t$ reduce to yield the generalized fractional integral operators (8) and (9), respectively (see [15, 16]). Further, setting $\mathtt{k}=1$, $g(t)=t$, $\lambda =\alpha $, $\sigma (0)=1$, and $w=0$ in (20) and (21) gives, respectively, the Riemann-Liouville fractional integrals (3) and (4).

The Hermite-Hadamard type inequalities in [22] have been generalized by Tunç *et al.* [24] who used the generalized k-fractional integral operators (20) and (21), which is recalled in the following theorem.

### Theorem 1

*Let* $\mathtt{k},\rho ,\lambda \in \mathbb{R}^{+}$, $w\in \mathbb{R}_{0}^{+}$, *and* $\sigma (m) \in \mathbb{R}^{+}$ $( m \in \mathbb{N}_{0} ) $ *be a bounded sequence*. *Also*, *let* $g :[a,b]\to \mathbb{R}$ *be an increasing and positive function on* $[a,b]$ *having a continuous derivative* $g{'}(x)$ *on* $(a,b)$. *If* *f* *is a convex function on* $[a,b]$, *then the following Hermite*-*Hadamard type inequalities for the generalized* k-*fractional integrals of* *f* *with respect to the function* *g* *on* $[a,b]$ *in* (20) *and* (21) *hold*:

26 $$ \begin{aligned}[b] f \biggl( \frac{a+b}{2} \biggr) \leq{}& \frac{1}{4\mathtt{k}(g(b)-g(a))^{\frac{ \lambda }{\mathtt{k}}}\mathcal{F}_{\rho ,\lambda +\mathtt{k}}^{\sigma ,\mathtt{k}} [w(g(b)-g(a))^{\rho }]} \\ & {} \times \bigl[ \mathcal{J}^{\sigma ,\mathtt{k},g}_{\rho , \lambda ,b{-};w}F(a)+ \mathcal{J}^{\sigma ,\mathtt{k},g}_{\rho ,\lambda ,a{+};w }F(b) \bigr] \leq \frac{f(a)+f(b)}{2}, \end{aligned} $$

*where* $F(x)$ *is defined as in* (28).

## Hermite-Hadamard type inequalities for fractional integral operators

We begin by recalling some notations given in [22]. Let *I* be an interval, such that $[a,b]\subset I$ $(0< a< b<\infty )$. Suppose that $f\in L^{\infty }[a,b]$ in such a way that $\mathcal{I} _{a{+},g}^{\alpha }f(x)$ and $\mathcal{I}_{b{-},g}^{\alpha }f(x)$ are well defined. We define the following functions:

27 $$ \tilde{f}(x):=f(a+b-x) $$

and

28 $$ F(x):=f(x)+f(a+b-x)=f(x)+\tilde{f}(x). $$

Also, the following notations will be used throughout this paper:

29 $$\begin{aligned}& \Delta_{\rho ,\lambda ,m}^{\sigma ,\mathtt{k},g}(s):= \biggl[ g(b)-g \biggl( \frac{s}{2}a+\frac{2-s}{2}b \biggr) \biggr] ^{\frac{\lambda }{ \mathtt{k}}+\rho m}; \end{aligned}$$

30 $$\begin{aligned}& \Omega_{\rho ,\lambda ,m}^{\sigma ,\mathtt{k},g}(s):= \biggl[ g \biggl( \frac{s}{2}b+ \frac{2-s}{2}a \biggr) -g(a) \biggr] ^{\frac{\lambda }{\mathtt{k}}+\rho m}; \end{aligned}$$

31 $$\begin{aligned}& \varphi_{\rho ,\lambda }^{\sigma ,\mathtt{k},g}(s):= \biggl[ g(b)-g \biggl( \frac{s}{2}a+\frac{2-s}{2}b \biggr) \biggr] ^{\frac{\lambda }{ \mathtt{k}}} \mathcal{F}_{\rho ,\lambda +\mathtt{k}}^{\sigma , \mathtt{k}} \biggl[ w \biggl( g(b)-g \biggl( \frac{s}{2}a+\frac{2-s}{2}b \biggr) \biggr) ^{\rho } \biggr] ; \end{aligned}$$

32 $$\begin{aligned}& \Phi_{\rho ,\lambda }^{\sigma ,\mathtt{k},g}(s):= \biggl[ g \biggl( \frac{s}{2}b+ \frac{2-s}{2}a \biggr) -g(a) \biggr] ^{\frac{\lambda }{\mathtt{k}}} \mathcal{F}_{\rho ,\lambda +\mathtt{k}}^{\sigma ,\mathtt{k}} \biggl[ w \biggl( g \biggl( \frac{s}{2}b+\frac{2-s}{2}a \biggr) -g(a) \biggr) ^{\rho } \biggr] . \end{aligned}$$

Taking $s=1$ in (31) and (32), respectively, gives

33 $$\begin{aligned}& \varphi_{\rho ,\lambda }^{\sigma ,\mathtt{k},g}(1)= \biggl[ g(b)-g \biggl( \frac{a+b}{2} \biggr) \biggr] ^{\frac{\lambda }{\mathtt{k}}} \mathcal{F}_{\rho ,\lambda +\mathtt{k}} ^{\sigma ,\mathtt{k}} \biggl[ w \biggl( g(b)-g \biggl( \frac{a+b}{2} \biggr) \biggr) ^{\rho } \biggr] ; \end{aligned}$$

34 $$\begin{aligned}& \Phi_{\rho ,\lambda }^{\sigma ,\mathtt{k},g}(1)= \biggl[ g \biggl( \frac{a+b}{2} \biggr) -g(a) \biggr] ^{\frac{\lambda }{\mathtt{k}}} \mathcal{F}_{\rho ,\lambda +\mathtt{k}} ^{\sigma ,\mathtt{k}} \biggl[ w \biggl( g \biggl( \frac{a+b}{2} \biggr) -g(a) \biggr) ^{\rho } \biggr] . \end{aligned}$$

The Hermite-Hadamard type inequalities for the generalized k-fractional integrals of a function with respect to another function in Theorem 1 can be modified as in the following theorem.

### Theorem 2

*Let* $\mathtt{k},\rho ,\lambda \in \mathbb{R}^{+}$, $w \in \mathbb{R}^{+}_{0}$, *and* $\sigma (m) \in \mathbb{R}^{+}$ $( m \in \mathbb{N}_{0} ) $ *be a bounded sequence*. *Also*, *let* $g :[a,b]\to \mathbb{R}$ *be an increasing and positive function on* $[a,b]$ *having a continuous derivative* $g{'}(x)$ *on* $(a,b)$. *If* *f* *is a convex function on* $[a,b]$, *then the following Hermite*-*Hadamard type inequalities for the generalized* k-*fractional integrals of* *f* *with respect to the function* *g* *on* $[a,b]$ *in* (20) *and* (21) *hold*:

35 $$ \begin{aligned}[b] f \biggl( \frac{a+b}{2} \biggr) \leq {}&\frac{1}{2\mathtt{k} [ \varphi_{\rho , \lambda }^{\sigma ,\mathtt{k},g}(1)+\Phi_{\rho ,\lambda }^{\sigma , \mathtt{k},g}(1) ] } \\ &{} \times \bigl[ \mathcal{J}^{\sigma ,\mathtt{k},g}_{\rho ,\lambda , \frac{a+b}{2}{-};w }F(a) + \mathcal{J}^{\sigma ,\mathtt{k},g}_{\rho , \lambda ,\frac{a+b}{2}{+};w }F(b) \bigr] \leq \frac{f(a)+f(b)}{2}, \end{aligned} $$

*where* $F(x)$ *is defined as in* (28).

### Proof

Since *f* is convex on $[a,b]$, we have

36 $$\begin{aligned}& f \biggl( \frac{x+y}{2} \biggr) \leq \frac{f(x)+f(y)}{2} \quad \bigl(x,y \in [a,b] \bigr). \end{aligned}$$

It is easy to see that

$$ \frac{s}{2}a+\frac{2-s}{2}b \quad \text{and} \quad \frac{2-s}{2}a+ \frac{s}{2}b \quad \bigl(s \in [0,1] \bigr) $$

belong to $[a,b]$. It follows from (36) that

37 $$\begin{aligned} 2f \biggl( \frac{a+b}{2} \biggr) \leq f \biggl( \frac{s}{2}a+\frac{2-s}{2}b \biggr) +f \biggl( \frac{2-s}{2}a+ \frac{s}{2}b \biggr) \quad \bigl(s \in [0,1] \bigr). \end{aligned}$$

Multiplying both sides of (37) by

$$ \frac{b-a}{2}\frac{g{'} ( \frac{s}{2}a+\frac{2-s}{2}b ) }{ [ g(b)-g ( \frac{s}{2}a+\frac{2-s}{2}b ) ] ^{1-\frac{ \lambda }{\mathtt{k} }}} \mathcal{F}_{\rho ,\lambda }^{\sigma , \mathtt{k} } \biggl[ w \biggl( g(b)-g \biggl( \frac{s}{2}a+\frac{2-s}{2}b \biggr) \biggr) ^{\rho } \biggr] $$

and integrating the resulting inequality on $[0,1]$ with respect to *s*, with the aid of (18), (19), (20), (27), (28), and (33), we obtain

38 $$ \begin{aligned}[b] 2\mathtt{k}f \biggl( \frac{a+b}{2} \biggr) \varphi_{\rho ,\lambda }^{ \sigma ,\mathtt{k},g}(1) & \leq \mathcal{J}^{\sigma ,\mathtt{k},g} _{\rho ,\lambda ,\frac{a+b}{2}{+};w }f(b)+\mathcal{J}^{\sigma , \mathtt{k},g}_{\rho ,\lambda ,\frac{a+b}{2}{+};w }\tilde{f}(b) \\ & = \mathcal{J}^{\sigma ,\mathtt{k},g}_{\rho ,\lambda ,\frac{a+b}{2}{+};w }F(b). \end{aligned} $$

Similarly, multiplying both sides of (37) by

$$ \frac{b-a}{2}\frac{g{'} ( \frac{s}{2}b+\frac{2-s}{2}a ) }{ [ g ( \frac{s}{2}b+\frac{2-s}{2}a ) -g(a) ] ^{1-\frac{ \lambda }{\mathtt{k}}}} \mathcal{F}_{\rho ,\lambda }^{\sigma , \mathtt{k}} \biggl[ w \biggl( g \biggl( \frac{s}{2}b+\frac{2-s}{2}a \biggr) -g(a) \biggr) ^{\rho } \biggr] $$

and integrating the resulting inequality on $[0,1]$ with respect to *s*, with the aid of (18), (19), (20), (27), (28), and (34), we get

39 $$ \begin{aligned}[b] 2\mathtt{k}f \biggl( \frac{a+b}{2} \biggr) \Phi_{\rho ,\lambda }^{\sigma , \mathtt{k},g}(1) &\leq \mathcal{J}^{\sigma ,\mathtt{k},g}_{\rho , \lambda ,\frac{a+b}{2}{-};w }f(a)+ \mathcal{J}^{\sigma ,\mathtt{k},g} _{\rho ,\lambda ,\frac{a+b}{2}{-};w }\tilde{f}(a) \\ &=\mathcal{J}^{ \sigma ,\mathtt{k},g}_{\rho ,\lambda ,\frac{a+b}{2}{-};w }F(a). \end{aligned} $$

From (38) and (39), we have

$$\begin{aligned}& 2\mathtt{k} f \biggl( \frac{a+b}{2} \biggr) \bigl[ \varphi_{\rho ,\lambda }^{\sigma ,\mathtt{k},g}(1)+ \Phi_{\rho ,\lambda }^{\sigma ,\mathtt{k},g}(1) \bigr] \\& \quad \leq \mathcal{J}^{\sigma ,\mathtt{k},g}_{\rho ,\lambda , \frac{a+b}{2}{-};w }F(a)+ \mathcal{J}^{\sigma ,\mathtt{k},g}_{\rho , \lambda ,\frac{a+b}{2}{+};w }F(b), \end{aligned}$$

which proves the first inequality in (35).

To prove the second inequality in (35), using the convexity of *f* on $[a,b]$, we obtain

$$ f \biggl( \frac{s}{2}a+\frac{2-s}{2}b \biggr) \leq \frac{s}{2} f(a)+ \frac{2-s}{2}f(b) \quad \bigl(s \in [0,1] \bigr) $$

and

$$ f \biggl( \frac{s}{2}b+\frac{2-s}{2}a \biggr) \leq \frac{s}{2}f(b)+ \frac{2-s}{2}f(a)\quad \bigl(s \in [0,1] \bigr). $$

By adding these inequalities, we get

40 $$\begin{aligned}& f \biggl( \frac{s}{2}a+\frac{2-s}{2}b \biggr) +f \biggl( \frac{2-s}{2}a+ \frac{s}{2}b \biggr) \leq f(a)+f(b) \quad \bigl(s \in [0,1] \bigr). \end{aligned}$$

Multiplying both sides of (40) by

$$ \frac{b-a}{2}\frac{g{'} ( \frac{s}{2}a+\frac{2-s}{2}b ) }{ [ g(b)-g ( \frac{s}{2}a+\frac{2-s}{2}b ) ] ^{1-\frac{ \lambda }{\mathtt{k}}}} \mathcal{F}_{\rho ,\lambda }^{\sigma , \mathtt{k}} \biggl[ w \biggl( g(b)-g \biggl( \frac{s}{2}a+\frac{2-s}{2}b \biggr) \biggr) ^{\rho } \biggr] $$

and integrating the resulting inequality on $[0,1]$ with respect to *s*, similar to the proof of the first inequality, we have

41 $$ \begin{aligned}[b] \mathcal{J}^{\sigma ,\mathtt{k},g}_{\rho ,\lambda ,\frac{a+b}{2}{+};w }f(b)+ \mathcal{J}^{\sigma ,\mathtt{k},g}_{\rho ,\lambda , \frac{a+b}{2}{+};w }\tilde{f}(b) &=\mathcal{J}^{\sigma ,\mathtt{k},g} _{\rho ,\lambda ,\frac{a+b}{2}{+};w }F(b) \\ &\leq \mathtt{k} \varphi_{\rho ,\lambda }^{\sigma ,\mathtt{k},g}(1) \bigl( f(a)+f(b) \bigr) . \end{aligned} $$

Similarly, multiplying both sides of (40) by

$$ \frac{b-a}{2}\frac{g{'} ( \frac{s}{2}b+\frac{2-s}{2}a ) }{ [ g ( \frac{s}{2}b+\frac{2-s}{2}a ) -g(a) ] ^{1-\frac{ \lambda }{\mathtt{k}}}} \mathcal{F}_{\rho ,\lambda }^{\sigma , \mathtt{k}} \biggl[ w \biggl( g \biggl( \frac{s}{2}b\frac{2-s}{2}a \biggr) -g(a) \biggr) ^{\rho } \biggr] $$

and integrating the resulting inequality on $[0,1]$ with respect to *s*, we obtain

42 $$ \begin{aligned}[b] \mathcal{J}^{\sigma ,\mathtt{k},g}_{\rho ,\lambda ,\frac{a+b}{2} ^{-};w }f(a)+ \mathcal{J}^{\sigma ,\mathtt{\mathtt{k}},g}_{\rho , \lambda ,\frac{a+b}{2}{-};w }\tilde{f}(a)& =\mathcal{J}^{\sigma , \mathtt{k},g}_{\rho ,\lambda ,\frac{a+b}{2}{-};w }F(a) \\ & \leq \mathtt{k}\Phi_{\rho ,\lambda }^{\sigma ,\mathtt{k},g}(1) \bigl( f(a)+f(b) \bigr) . \end{aligned} $$

Adding (41) and (42), we have

43 $$ \begin{aligned}[b] & \mathcal{J}^{\sigma ,\mathtt{k},g}_{\rho ,\lambda ,\frac{a+b}{2} {-};w }F(a) +\mathcal{J}^{\sigma ,\mathtt{k},g}_{\rho ,\lambda , \frac{a+b}{2}{+};w }F(b) \\ & \quad \leq \mathtt{k} \bigl[ \varphi_{\rho ,\lambda }^{\sigma , \mathtt{k},g}(1)+ \Phi_{\rho ,\lambda }^{\sigma ,\mathtt{k},g}(1) \bigr] \bigl( f(a)+f(b) \bigr) , \end{aligned} $$

which proves the second inequality in (35). Hence this completes the proof. □

Setting $\mathtt{k}=1$ in Theorem 2, we get a little simpler inequalities asserted by the following corollary.

### Corollary 1

*Let* $\rho ,\lambda \in \mathbb{R}^{+}$, $w \in \mathbb{R}^{+}_{0}$, *and* $\sigma (m) \in \mathbb{R}^{+}$ $( m\in \mathbb{N}_{0} ) $ *be a bounded sequence*. *Also*, *let* $g :[a,b]\to \mathbb{R}$ *be an increasing and positive function on* $[a,b]$ *having a continuous derivative* $g{'}(x)$ *on* $(a,b)$. *If* *f* *is a convex function on* $[a,b]$, *then the following Hermite*-*Hadamard type inequalities for the generalized fractional integrals of* *f* *with respect to the function* *g* *on* $[a,b]$ *in* (20) *and* (21) *with* $\mathtt{k}=1$ *hold*:

44 $$ \begin{aligned}[b] f \biggl( \frac{a+b}{2} \biggr) & \leq \frac{1}{2 [ \varphi_{\rho ,\lambda } ^{\sigma ,1,g}(1)+\Phi_{\rho ,\lambda }^{\sigma ,1,g}(1) ] } \bigl[ \mathcal{J}^{\sigma ,1,g}_{\rho ,\lambda ,\frac{a+b}{2}{-};w }F(a) + \mathcal{J}^{\sigma ,1,g}_{\rho ,\lambda ,\frac{a+b}{2}{+};w }F(b) \bigr] \\ &\leq \frac{f(a)+f(b)}{2}, \end{aligned} $$

*where* $F(x)$ *is defined as in* (28).

Further, choosing $\lambda =\alpha $, $\sigma (0)=1$ and $w=0$ in Corollary 1, we get simpler inequalities in the following corollary, which are a modification of the Hermite-Hadamard inequalities given in [22].

### Corollary 2

*Let* $\alpha \in \mathbb{R}^{+}$ *and* $g :[a,b]\to \mathbb{R}$ *be an increasing and positive function on* $[a,b]$ *having a continuous derivative* $g{'}(x)$ *on* $(a,b)$. *If* *f* *is a convex function on* $[a,b]$, *then the following Hermite*-*Hadamard type inequalities for the generalized fractional integrals of* *f* *with respect to the function* *g* *on* $[a,b]$ *in* (14) *and* (15) *hold*:

45 $$ \begin{aligned}[b] f \biggl( \frac{a+b}{2} \biggr) & \leq \frac{\Gamma (\alpha +1)}{2 ( [ g(b)-g ( \frac{a+b}{2} ) ] ^{\alpha }+ [ g ( \frac{a+b}{2} ) -g(a) ] ^{\alpha } )} \bigl[ \mathcal{I}_{\frac{a+b}{2}{+};g}^{\alpha }F(b)+ \mathcal{I}_{\frac{a+b}{2}{-};g}^{\alpha }F(a) \bigr] \\ &\leq \frac{f(a)+f(b)}{2}, \end{aligned} $$

*where* $F(x)$ *is defined as in* (28).

It is remarked in passing that choosing $g(t)=t$ in Corollary 1 yields the same result as in [21], Corollary 1.

## Main results

We begin by presenting an integral formula involving the functions (31) and (32), which is asserted by the following lemma.

### Lemma 2

*Let* $\mathtt{k},\rho ,\lambda \in \mathbb{R}^{+}$, $w \in \mathbb{R}$, *and* $\sigma (m) \in \mathbb{R}^{+}$ $( m\in \mathbb{N}_{0} ) $ *be a bounded sequence*. *Also*, *let* $g :[a,b] \to \mathbb{R}$ *be an increasing and positive function on* $[a,b]$ *having a continuous derivative* $g{'}(x)$ *on* $(a,b)$. *Further*, *let* $f :[a,b] \to \mathbb{R}$ *be a differentiable mapping on* $(a,b)$ $(a< b)$ *and* $f' \in L[a,b]$. *Then*

46 $$ \begin{aligned}[b] &\frac{1}{2\mathtt{k}} \bigl[ \mathcal{J}^{\sigma ,\mathtt{k},g}_{ \rho ,\lambda ,\frac{a+b}{2}{+};w }F(b) +\mathcal{J}^{\sigma , \mathtt{k},g}_{\rho ,\lambda ,\frac{a+b}{2}{-};w}F(a) \bigr] - \bigl( \varphi _{\rho ,\lambda }^{\sigma ,\mathtt{k},g}(1)+\Phi_{\rho ,\lambda }^{ \sigma ,\mathtt{k},g}(1) \bigr) f \biggl( \frac{a+b}{2} \biggr) \\ &\quad = \frac{b-a}{4} \biggl[ \int_{0}^{1} \bigl( \varphi_{\rho ,\lambda }^{ \sigma ,\mathtt{k},g}(s) +\Phi_{\rho ,\lambda }^{\sigma ,\mathtt{k},g}(s) \bigr) f {'} \biggl( \frac{s}{2}a+\frac{2-s}{2}b \biggr) \,ds \\ &\qquad {} - \int_{0}^{1} \bigl( \varphi_{\rho ,\lambda }^{\sigma , \mathtt{k},g}(s) +\Phi_{\rho ,\lambda }^{\sigma ,\mathtt{k},g}(s) \bigr) f {'} \biggl( \frac{s}{2}b+\frac{2-s}{2}a \biggr) \,ds \biggr], \end{aligned} $$

*where* $\varphi_{\rho ,\lambda }^{\sigma ,\mathtt{k},g}(s)$ *and* $\Phi_{\rho ,\lambda }^{\sigma ,\mathtt{k},g}(s)$ *are given as in* (31) *and* (32).

### Proof

Using (20) and changing the variable

$$ t=\frac{s}{2}a+\frac{2-s}{2}b \quad (0\leq s \leq 1), $$

we find

47 $$ \begin{aligned}[b] \mathcal{J}^{\sigma ,\mathtt{k},g}_{\rho ,\lambda ,\frac{a+b}{2} {+};w }F(b)={}& \frac{b-a}{2} \int_{0}^{1}\frac{g{'} ( \frac{s}{2}a+ \frac{2-s}{2}b ) }{ [ g(b)-g ( \frac{s}{2}a+\frac{2-s}{2}b ) ] ^{1-\frac{\lambda }{\mathtt{k}}}} \\ & {} \times \mathcal{F}_{\rho ,\lambda }^{\sigma ,\mathtt{k}} \biggl[ w \biggl( g(b)-g \biggl( \frac{s}{2}a+\frac{2-s}{2}b \biggr) \biggr) ^{\rho } \biggr] F \biggl( \frac{s}{2}a+ \frac{2-s}{2}b \biggr) \,ds. \end{aligned} $$

Integrating (47) by parts, we have

48 $$\begin{aligned} \begin{aligned}[b] &\mathcal{J}^{\sigma ,\mathtt{\mathtt{k}},g}_{\rho ,\lambda , \frac{a+b}{2}{+};w }F(b)\\ &\quad = \mathtt{k} \biggl[ g(b)-g \biggl(\frac{a+b}{2} \biggr) \biggr] ^{\frac{\lambda }{\mathtt{k}}} \mathcal{F}_{\rho ,\lambda +\mathtt{k}} ^{\sigma ,\mathtt{k}} \biggl[ w \biggl( g(b)-g \biggl( \frac{a+b}{2} \biggr) \biggr) ^{\rho } \biggr] F \biggl( \frac{a+b}{2} \biggr) \\ &\qquad {} +\frac{b-a}{2} \mathtt{k} \int_{0}^{1} \biggl[ g(b)-g \biggl( \frac{s}{2}a+\frac{2-s}{2}b \biggr) \biggr] ^{\frac{\lambda }{\mathtt{k}}} \mathcal{F}_{\rho ,\lambda +\mathtt{k}} ^{\sigma ,\mathtt{k}} \biggl[ w \biggl( g(b)-g \biggl( \frac{s}{2}a+ \frac{2-s}{2}b \biggr) \biggr) ^{\rho } \biggr] \\ & \qquad {} \times F{'} \biggl( \frac{s}{2}a+ \frac{2-s}{2}b \biggr) \,ds. \end{aligned} \end{aligned}$$

Similarly, using (21) and changing the variable

$$ t=\frac{s}{2}b+\frac{2-s}{2}a \quad (0\leq s \leq 1), $$

and integrating the resulting identity by parts, we have

49 $$\begin{aligned} \begin{aligned}[b] &\mathcal{J}^{\sigma ,\mathtt{k},g}_{\rho ,\lambda ,\frac{a+b}{2} {-};w}F(a)\\ &\quad = \mathtt{k} \biggl[ g \biggl( \frac{a+b}{2} \biggr) -g(a) \biggr] ^{\frac{\lambda }{\mathtt{k}}}\mathcal{F}_{\rho ,\lambda +\mathtt{k}} ^{\sigma ,\mathtt{k}} \biggl[ w \biggl( g \biggl( \frac{a+b}{2} \biggr) -g(a) \biggr) ^{\rho } \biggr] F \biggl( \frac{a+b}{2} \biggr) \\ &\qquad {} -\frac{b-a}{2} \mathtt{k} \int_{0}^{1} \biggl( g \biggl( \frac{s}{2}b+ \frac{2-s}{2}a \biggr) -g(a) \biggr) ^{\frac{\lambda }{\mathtt{k}}} \mathcal{F}_{\rho ,\lambda +\mathtt{k}} ^{\sigma ,\mathtt{k}} \biggl[ w \biggl( g \biggl( \frac{s}{2}b+ \frac{2-s}{2}a \biggr) -g(a) \biggr) ^{\rho } \biggr] \\ &\qquad {} \times F{'} \biggl( \frac{s}{2}b+ \frac{2-s}{2}a \biggr) \,ds. \end{aligned} \end{aligned}$$

Using (28) to add (48) and (49), we obtain

50 $$\begin{aligned} \begin{aligned}[b] & \frac{1}{2\mathtt{k}} \bigl[ \mathcal{J}^{\sigma ,\mathtt{\mathtt{k}},g} _{\rho ,\lambda ,\frac{a+b}{2}{+};w }F(b) + \mathcal{J}^{\sigma , \mathtt{k},g}_{\rho ,\lambda ,\frac{a+b}{2}{-};w}F(a) \bigr] \\ &\quad = \biggl[ g(b)-g \biggl(\frac{a+b}{2} \biggr) \biggr] ^{\frac{\lambda }{\mathtt{k}}} \mathcal{F}_{\rho ,\lambda +\mathtt{k}}^{\sigma ,\mathtt{k}} \biggl[ w \biggl( g(b)-g \biggl( \frac{a+b}{2} \biggr) \biggr) ^{\rho } \biggr] f \biggl( \frac{a+b}{2} \biggr) \\ &\qquad {}+ \biggl[ g \biggl( \frac{a+b}{2} \biggr) -g(a) \biggr] ^{\frac{\lambda }{ \mathtt{k}}} \mathcal{F}_{\rho ,\lambda +\mathtt{k}}^{\sigma , \mathtt{k}} \biggl[ w \biggl( g \biggl( \frac{a+b}{2} \biggr) -g(a) \biggr) ^{ \rho } \biggr] f \biggl( \frac{a+b}{2} \biggr) \\ &\qquad {} +\frac{b-a}{4} \int_{0} ^{1} \biggl[ g(b)-g \biggl( \frac{s}{2}a+\frac{2-s}{2}b \biggr) \biggr] ^{\frac{ \lambda }{\mathtt{k}}} \mathcal{F}_{\rho ,\lambda +\mathtt{k}}^{ \sigma ,\mathtt{k}} \biggl[ w \biggl( g(b)-g \biggl( \frac{s}{2}a+ \frac{2-s}{2}b \biggr) \biggr) ^{\rho } \biggr] \\ & \qquad {} \times F{'} \biggl( \frac{s}{2}a+ \frac{2-s}{2}b \biggr) \,ds \\ &\qquad {}-\frac{b-a}{4} \int_{0}^{1} \biggl( g \biggl( \frac{s}{2}b+ \frac{2-s}{2}a \biggr) -g(a) \biggr) ^{\frac{\lambda }{\mathtt{k}}} \mathcal{F}_{\rho ,\lambda +\mathtt{k}} ^{\sigma ,\mathtt{k}} \biggl[ w \biggl( g \biggl( \frac{s}{2}b+ \frac{2-s}{2}a \biggr) -g(a) \biggr) ^{\rho } \biggr] \\ & \qquad {} \times F{'} \biggl( \frac{s}{2}b+ \frac{2-s}{2}a \biggr) \,ds. \end{aligned} \end{aligned}$$

Considering $F{'}(x)=f{'}(x)-f{'}(a+b-x)$ and applying (31)-(34) to (50), we obtain the desired identity (46). □

Setting $\mathtt{k}=1$ in Lemma 2, we obtain an identity asserted by the following corollary.

### Corollary 3

*Let* $\rho ,\lambda \in \mathbb{R}^{+}$, $w \in \mathbb{R}$, *and* $\sigma (m) \in \mathbb{R}^{+}$ $( m\in \mathbb{N}_{0} ) $ *be a bounded sequence*. *Also*, *let* $g :[a,b]\to \mathbb{R}$ *be an increasing and positive function on* $[a,b]$ *having a continuous derivative* $g{'}(x)$ *on* $(a,b)$. *Further*, *let* $f :[a,b]\to \mathbb{R}$ *be a differentiable mapping on* $(a,b)$ $(a< b)$ *and* $f' \in L[a,b]$. *Then*

51 $$ \begin{aligned}[b] & \mathcal{J}^{\sigma ,1,g}_{\rho ,\lambda ,\frac{a+b}{2}{+};w }F(b) + \mathcal{J}^{\sigma ,1,g}_{\rho ,\lambda ,\frac{a+b}{2}{-};w}F(a) -2 \bigl( \varphi_{\rho ,\lambda }^{\sigma ,1,g}(1)+ \Phi_{\rho ,\lambda }^{\sigma ,1,g}(1) \bigr) f \biggl( \frac{a+b}{2} \biggr) \\ & \quad =\frac{b-a}{2} \biggl[ \int_{0}^{1} \bigl( \varphi_{\rho , \lambda }^{\sigma ,1,g}(s) +\Phi_{\rho ,\lambda }^{\sigma ,1,g}(s) \bigr) f {'} \biggl( \frac{s}{2}a+\frac{2-s}{2}b \biggr) \,ds \\ & \qquad {} - \int_{0}^{1} \bigl( \varphi_{\rho ,\lambda }^{\sigma ,1,g}(s) +\Phi_{\rho ,\lambda }^{\sigma ,1,g}(s) \bigr) f{'} \biggl( \frac{s}{2}b+ \frac{2-s}{2}a \biggr) \,ds \biggr], \end{aligned} $$

*where* $\varphi_{\rho ,\lambda }^{\sigma ,1,g}(s)$ *and* $\Phi_{\rho ,\lambda }^{\sigma ,1,g}(s)$ *are given as in* (31) *and* (32).

Choosing $\mathtt{k}=1$, $\lambda =\alpha $, $\sigma (0)=1$, and $w=0$ in Lemma 2 yields an interesting identity asserted by the following corollary.

### Corollary 4

*Let* $\alpha \in \mathbb{C}$ *with* $\Re (\alpha )>1$. *Also*, *let* $g :[a,b]\to \mathbb{R}$ *be an increasing and positive function on* $[a,b]$ *having a continuous derivative* $g{'}(x)$ *on* $(a,b)$. *Further*, *let* $f :[a,b]\to \mathbb{R}$ *be a differentiable mapping on* $(a,b)$ $(a< b)$ *and* $f' \in L[a,b]$. *Then*

52 $$ \begin{aligned}[b] &\frac{\Gamma (\alpha +1)}{2} \bigl[ \mathcal{I}_{\frac{a+b}{2}{+};g} ^{\alpha }F(b)+\mathcal{I}_{\frac{a+b}{2}{-};g}^{\alpha }F(a) \bigr] \\ & \qquad {}- \biggl( \biggl[ g(b)-g \biggl( \frac{a+b}{2} \biggr) \biggr] ^{\alpha } + \biggl[ g \biggl( \frac{a+b}{2} \biggr) -g(a) \biggr] ^{\alpha } \biggr)f \biggl( \frac{a+b}{2} \biggr) \\ &\quad =\frac{b-a}{4} \biggl\{ \int_{0}^{1} \biggl( \biggl[ g(b)-g \biggl( \frac{s}{2}a+\frac{2-s}{2}b \biggr) \biggr] ^{\alpha } + \biggl[ \biggl( g \biggl( \frac{s}{2}b+\frac{2-s}{2}a \biggr) -g(a) \biggr) \biggr] ^{\alpha } \biggr) \\ & \qquad {} \times f{'} \biggl( \frac{s}{2}a+ \frac{2-s}{2}b \biggr) \,ds \\ & \qquad {}- \int_{0}^{1} \biggl( \biggl[ g(b)-g \biggl( \frac{s}{2}a+ \frac{2-s}{2}b \biggr) \biggr] ^{\alpha } + \biggl[ \biggl( g \biggl( \frac{s}{2}b+ \frac{2-s}{2}a \biggr) -g(a) \biggr) \biggr] ^{\alpha } \biggr) \\ & \qquad {} \times f{'} \biggl( \frac{s}{2}b+ \frac{2-s}{2}a \biggr) \, ds \biggr\} , \end{aligned} $$

*where* $\mathcal{I}_{a+;g}^{\alpha }f(x)$ *and* $\mathcal{I}_{b-;g}^{ \alpha }f(x)$ *are given in* (14) *and* (15).

### Remark 1

Setting $\mathtt{k}=1$ and $g(t)=t$ in Lemma 2 gives the same result as in Lemma 1.

Taking $\mathtt{k}=1$, $\lambda =\alpha $, $g(t)=t$, $\sigma (0)=1$ and $w=0$ in Lemma 2 yields the same result as in [11], Lemma 3.

### Theorem 3

*Let* $\mathtt{k},\rho ,\lambda \in \mathbb{R}^{+}$, $w \in \mathbb{R}_{0}^{+}$, *and* $\sigma (m) \in \mathbb{R}^{+}$ $( m \in \mathbb{N}_{0} ) $ *be a bounded sequence*. *Also*, *let* $g :[a,b]\to \mathbb{R}$ *be an increasing and positive function on* $[a,b]$ *having a continuous derivative* $g{'}(x)$ *on* $(a,b)$. *Further*, *let* $f :[a,b]\to \mathbb{R}$ *be a differentiable mapping on* $(a,b)$ $(a< b)$ *such that* $f' \in L[a,b]$ *and* $\vert f'\vert $ *is a convex function on* $[a,b]$. *Then*

53 $$ \begin{aligned}[b] & \biggl\vert \frac{1}{2\mathtt{k}} \bigl[ \mathcal{J}^{\sigma ,\mathtt{k},g} _{\rho ,\lambda ,\frac{a+b}{2}{+};w }F(b) +\mathcal{J}^{\sigma , \mathtt{k},g}_{\rho ,\lambda ,\frac{a+b}{2}{-};w}F(a) \bigr] - \bigl( \varphi _{\rho ,\lambda }^{\sigma ,\mathtt{k},g}(1)+\Phi_{\rho ,\lambda }^{ \sigma ,\mathtt{k},g}(1) \bigr) f \biggl( \frac{a+b}{2} \biggr) \biggr\vert \\ & \quad \leq \frac{b-a}{4} \bigl( \mathcal{F}_{\rho ,\lambda + \mathtt{k}}^{\sigma_{1},\mathtt{k}} [w] \bigr) \bigl( \bigl\vert f{'}(a) \bigr\vert + \bigl\vert f{'}(b) \bigr\vert \bigr) , \end{aligned} $$

*where the notations are given as above and*

54 $$ \sigma_{1}(m):=\sigma (m) \int_{0}^{1} \bigl( \Delta_{\rho ,\lambda ,m} ^{\sigma ,\mathtt{k},g}(s) +\Omega_{\rho ,\lambda ,m}^{\sigma , \mathtt{k},g}(s) \bigr) \,ds \quad ( m \in \mathbb{N}_{0} ) . $$

### Proof

Here and in the following, let $\mathcal{L}$ be the left side of the equality in (46). Since *g* is increasing on $[a,b]$ and $\vert f'\vert $ is a convex function on $[a,b]$, in view of (19), (31) and (32), we find from Lemma 2 that

55 $$ \begin{aligned}[b] \vert \mathcal{L}\vert \leq{}& \frac{b-a}{4}\sum_{m=0}^{\infty } \frac{\sigma (m) w^{m}}{\mathtt{k} \Gamma_{\mathtt{k}}(\rho m \mathtt{k}+\lambda + \mathtt{k})} \\ & {} \times \biggl\{ \int_{0}^{1} \biggl[ g(b)-g \biggl( \frac{s}{2}a+ \frac{2-s}{2}b \biggr) \biggr] ^{\frac{\lambda }{\mathtt{k}}+\rho m} \biggl( \frac{s}{2} \bigl\vert f{'}(a) \bigr\vert + \frac{2-s}{2} \bigl\vert f{'}(b) \bigr\vert \biggr)\,ds \\ & {}+ \int_{0}^{1} \biggl[ g(b)-g \biggl( \frac{s}{2}a+\frac{2-s}{2}b \biggr) \biggr] ^{\frac{\lambda }{\mathtt{k}}+\rho m} \biggl( \frac{s}{2} \bigl\vert f{'}(b) \bigr\vert + \frac{2-s}{2} \bigl\vert f{'}(a) \bigr\vert \biggr)\,ds \\ & {}+ \int_{0}^{1} \biggl( g \biggl( \frac{s}{2}b+ \frac{2-s}{2}a \biggr) -g(a) \biggr) ^{\frac{\lambda }{\mathtt{k}}+\rho m} \biggl( \frac{s}{2} \bigl\vert f{'}(b) \bigr\vert + \frac{2-s}{2} \bigl\vert f{'}(a) \bigr\vert \biggr)\,ds \\ & {}+ \int_{0}^{1} \biggl( g \biggl( \frac{s}{2}b+ \frac{2-s}{2}a \biggr) -g(a) \biggr) ^{\frac{\lambda }{\mathtt{k}}+\rho m} \biggl( \frac{s}{2} \bigl\vert f{'}(a) \bigr\vert + \frac{2-s}{2} \bigl\vert f{'}(b) \bigr\vert \biggr)\,ds \biggr\} . \end{aligned} $$

Since

$$ \frac{s}{2} \bigl\vert f{'}(b) \bigr\vert + \frac{2-s}{2} \bigl\vert f{'}(a) \bigr\vert + \frac{s}{2} \bigl\vert f{'}(a) \bigr\vert + \frac{2-s}{2} \bigl\vert f{'}(b) \bigr\vert = \bigl\vert f{'}(a) \bigr\vert + \bigl\vert f{'}(b) \bigr\vert , $$

using (29) and (30), we have

$$ \begin{aligned}[b] \vert \mathcal{L}\vert \leq{}& \frac{b-a}{4} \bigl( \bigl\vert f{'}(a) \bigr\vert + \bigl\vert f{'}(b) \bigr\vert \bigr) \sum_{m=0}^{\infty } \frac{\sigma (m)w^{m}}{\mathtt{k} \Gamma_{\mathtt{k}}(\rho m \mathtt{k}+\lambda +\mathtt{k})} \\ &{}\times \int_{0}^{1} \biggl[ \biggl( g(b)-g \biggl( \frac{s}{2}a+\frac{2-s}{2}b \biggr) \biggr) ^{\frac{\lambda }{\mathtt{k}}+\rho m} + \biggl( g \biggl( \frac{s}{2}b+ \frac{2-s}{2}a \biggr) -g(a) \biggr) ^{\frac{\lambda }{\mathtt{k}}+\rho m} \biggr] \,ds \\ ={}&\frac{b-a}{4} \bigl( \bigl\vert f^{\prime}(a) \bigr\vert + \bigl\vert f^{\prime}(b) \bigr\vert \bigr) \sum _{m=0} ^{\infty }\frac{w^{m}}{\mathtt{k} \Gamma_{\mathtt{k}}(\rho m \mathtt{k}+\lambda +\mathtt{k})} \sigma (m) \int_{0}^{1} \bigl( \Delta_{ \rho ,\lambda ,m}^{\sigma ,\mathtt{k},g}(s) +\Omega_{\rho ,\lambda ,m} ^{\sigma ,\mathtt{k},g}(s) \bigr)\,ds \\ ={}&\frac{b-a}{4} \bigl( \bigl\vert f^{\prime}(a) \bigr\vert + \bigl\vert f ^{\prime}(b) \bigr\vert \bigr) \mathcal{F}_{\rho ,\lambda +\mathtt{k}}^{\sigma_{1}, \mathtt{k}} [w]. \end{aligned} $$

This completes the proof. □

Choosing $\mathtt{k}=1$, $\lambda =\alpha $, $\sigma (0)=1$, and $w=0$ in Theorem 3, we obtain an interesting inequality involving the generalized fractional integrals (14) and (15), which is asserted by the following corollary.

### Corollary 5

*Let* $\alpha \in \mathbb{R}^{+}$ *and* $g :[a,b]\to \mathbb{R}$ *be an increasing and positive function on* $[a,b]$ *having a continuous derivative* $g{'}(x)$ *on* $(a,b)$. *Also*, *let* $f :[a,b]\to \mathbb{R}$ *be a differentiable mapping on* $(a,b)$ $(a< b)$ *such that* $f' \in L[a,b]$ *and* $\vert f'\vert $ *is a convex function on* $[a,b]$. *Then*

56 $$ \begin{aligned}[b] & \biggl\vert \frac{\Gamma (\alpha +1)}{2} \bigl[ \mathcal{I}_{\frac{a+b}{2} {+};g}^{\alpha }F(b)+\mathcal{I}_{\frac{a+b}{2}{-};g}^{\alpha }F(a) \bigr] -\eta^{\alpha }_{a,b;g} f \biggl( \frac{a+b}{2} \biggr) \biggr\vert \\ & \quad \leq \frac{b-a}{4}\Upsilon_{a,b;g}^{\alpha } \bigl( \bigl\vert f {'}(a) \bigr\vert + \bigl\vert f{'}(b) \bigr\vert \bigr) , \end{aligned} $$

*where*

57 $$ \eta^{\alpha }_{a,b;g}:= \biggl( \biggl[ g(b)-g \biggl( \frac{a+b}{2} \biggr) \biggr] ^{\alpha }+ \biggl[ g \biggl( \frac{a+b}{2} \biggr) -g(a) \biggr] ^{\alpha } \biggr) $$

*and*

58 $$ \Upsilon_{a,b;g}^{\alpha }:= \int_{0}^{1} \biggl[ \biggl( g(b)-g \biggl( \frac{s}{2}a+ \frac{2-s}{2}b \biggr) \biggr) ^{\alpha } + \biggl( g \biggl( \frac{s}{2}b+ \frac{2-s}{2}a \biggr) -g(a) \biggr) ^{\alpha } \biggr] \,ds. $$

### Remark 2

Setting $k=1$ and $g(t)=t$ in Theorem 3 gives the same result as in [21], Corollary 3.

The special case of Theorem 3 when $\mathtt{k}=1$, $\lambda = \alpha $, $\sigma (0)=1$, and $w=0$ is seen to correspond with the result obtained by setting $q=1$ in [11], Theorem 5.

### Theorem 4

*Let* $\mathtt{k},\rho ,\lambda \in \mathbb{R}^{+}$, $w \in \mathbb{R}_{0}^{+}$, *and* $\sigma (m) \in \mathbb{R}^{+}$ $( m \in \mathbb{N}_{0} ) $ *be a bounded sequence*. *Also*, *let* $g :[a,b]\to \mathbb{R}$ *be an increasing and positive function on* $[a,b]$ *having a continuous derivative* $g{'}(x)$ *on* $(a,b)$. *Further*, *let* $f :[a,b]\to \mathbb{R}$ *be a differentiable mapping on* $(a,b)$ $(a< b)$ *such that* $\vert f{'}\vert ^{q}$ *is convex for* $q>1$ *with* $\frac{1}{p}+\frac{1}{q}=1$. *Then*

59 $$ \begin{aligned}[b] & \biggl\vert \frac{1}{2\mathtt{k}} \bigl[ \mathcal{J}^{\sigma ,\mathtt{k},g} _{\rho ,\lambda ,\frac{a+b}{2}{+};w }F(b) +\mathcal{J}^{\sigma , \mathtt{k},g}_{\rho ,\lambda ,\frac{a+b}{2}{-};w}F(a) \bigr] - \bigl( \varphi _{\rho ,\lambda }^{\sigma ,\mathtt{k},g}(1)+\Phi_{\rho ,\lambda }^{ \sigma ,\mathtt{k},g}(1) \bigr) f \biggl( \frac{a+b}{2} \biggr) \biggr\vert \\ &\quad \leq \frac{b-a}{4}\mathcal{F}_{\rho ,\lambda +\mathtt{k}}^{\sigma_{2}, \mathtt{k}} [w] \biggl[ \biggl( \frac{1}{4} \bigl\vert f{'}(a) \bigr\vert ^{q}+\frac{3}{4} \bigl\vert f {'}(b) \bigr\vert ^{q} \biggr) ^{\frac{1}{q}} + \biggl( \frac{3}{4} \bigl\vert f{'}(a) \bigr\vert ^{q}+ \frac{1}{4} \bigl\vert f{'}(b) \bigr\vert ^{q} \biggr) ^{\frac{1}{q}} \biggr] , \end{aligned} $$

*where the notations are given as above and*

$$ \sigma_{2}(m):=\sigma (m) \biggl( \biggl[ \int_{0}^{1} \bigl( \Delta_{\rho ,\lambda ,m}^{\sigma ,\mathtt{k},g}(s) \bigr) ^{p}\,ds \biggr] ^{\frac{1}{p}} + \biggl[ \int_{0}^{1} \bigl( \Omega_{\rho ,\lambda ,m}^{\sigma ,\mathtt{k},g}(s) \bigr) ^{p}\,ds \biggr] ^{\frac{1}{p}} \biggr) . $$

### Proof

Using convexity of $\vert f{'}\vert ^{q}$ and Hölder’s inequality in Lemma 2, we have

$$ \begin{aligned}[b] & \vert \mathcal{L}\vert \leq \frac{b-a}{4} \sum_{m=0}^{\infty }\frac{\sigma (m) w^{m}}{\mathtt{k} \Gamma_{\mathtt{k}}(\rho m \mathtt{k}+\lambda + \mathtt{k})} \Biggl( \sum_{j=1}^{4} \mathcal{I}_{j} \Biggr) , \end{aligned} $$

where

$$\begin{aligned}& \begin{aligned} \mathcal{I}_{1}:={}& \biggl( \int_{0}^{1} \biggl[ g(b)-g \biggl( \frac{s}{2}a+ \frac{2-s}{2}b \biggr) \biggr] ^{p(\frac{\lambda }{\mathtt{k}}+\rho m)}\,ds \biggr)^{\frac{1}{p}}\\ &{}\times \biggl( \int_{0}^{1} \biggl( \frac{s}{2} \bigl\vert f{'}(a) \bigr\vert ^{q}+ \frac{2-s}{2} \bigl\vert f{'}(b) \bigr\vert ^{q} \biggr)\,ds \biggr)^{\frac{1}{q}}, \end{aligned} \\& \begin{aligned} \mathcal{I}_{2}:={}& \biggl( \int_{0}^{1} \biggl[ g(b)-g \biggl( \frac{s}{2}b+ \frac{2-s}{2}a \biggr) \biggr] ^{p(\frac{\lambda }{\mathtt{k}}+\rho m)}\,ds \biggr)^{\frac{1}{p}}\\ &{}\times \biggl( \int_{0}^{1} \biggl( \frac{s}{2} \bigl\vert f{'}(b) \bigr\vert ^{q}+ \frac{2-s}{2} \bigl\vert f{'}(a) \bigr\vert ^{q} \biggr)\,ds \biggr)^{\frac{1}{q}}, \end{aligned} \\& \begin{aligned} \mathcal{I}_{3}:={}& \biggl( \int_{0}^{1} \biggl( g \biggl( \frac{s}{2}b+ \frac{2-s}{2}a \biggr) -g(a) \biggr) ^{p(\frac{\lambda }{\mathtt{k}}+ \rho m)}\,ds \biggr)^{\frac{1}{p}}\\ &{}\times \biggl( \int_{0}^{1} \biggl( \frac{s}{2} \bigl\vert f {'}(a) \bigr\vert ^{q}+\frac{2-s}{2} \bigl\vert f{'}(b) \bigr\vert ^{q} \biggr)\,ds \biggr)^{\frac{1}{q}}, \end{aligned} \\& \begin{aligned} \mathcal{I}_{4}:={}& \biggl( \int_{0}^{1} \biggl( g \biggl( \frac{s}{2}b+ \frac{2-s}{2}a \biggr) -g(a) \biggr) ^{p(\frac{\lambda }{\mathtt{k}}+ \rho m)}\,ds \biggr)^{\frac{1}{p}}\\ &{}\times \biggl( \int_{0}^{1} \biggl( \frac{s}{2} \bigl\vert f {'}(b) \bigr\vert ^{q}+\frac{2-s}{2} \bigl\vert f{'}(a) \bigr\vert ^{q} \biggr)\,ds \biggr)^{\frac{1}{q}}. \end{aligned} \end{aligned}$$

We thus have

$$ \begin{aligned}[b] \vert \mathcal{L}\vert \leq{}& \frac{b-a}{4} \sum_{m=0}^{\infty }\frac{\sigma (m) w^{m}}{\mathtt{k} \Gamma_{\mathtt{k}}(\rho m \mathtt{k}+\lambda + \mathtt{k})} \biggl[ \biggl( \int_{0}^{1} \bigl( \Delta_{\rho ,\lambda ,m} ^{\sigma ,k,g}(s) \bigr) ^{p}\,ds \biggr) ^{\frac{1}{p}} + \biggl( \int_{0} ^{1} \bigl( \Omega_{\rho ,\lambda ,m}^{\sigma ,k,g}(s) \bigr) ^{p}\,ds \biggr) ^{\frac{1}{p}} \biggr] \\ & {} \times \biggl[ \biggl( \frac{1}{4} \bigl\vert f{'}(a) \bigr\vert ^{q}+\frac{3}{4} \bigl\vert f {'}(b) \bigr\vert ^{q} \biggr) ^{\frac{1}{q}} + \biggl( \frac{3}{4} \bigl\vert f{'}(a) \bigr\vert ^{q}+ \frac{1}{4} \bigl\vert f{'}(b) \bigr\vert ^{q} \biggr) ^{\frac{1}{q}} \biggr] \\ ={}& \frac{b-a}{4}\mathcal{F}_{\rho ,\lambda +\mathtt{k}}^{\sigma_{2}, \mathtt{k}} [w] \biggl[ \biggl( \frac{1}{4} \bigl\vert f{'}(a) \bigr\vert ^{q}+\frac{3}{4} \bigl\vert f {'}(b) \bigr\vert ^{q} \biggr) ^{\frac{1}{q}} + \biggl( \frac{3}{4} \bigl\vert f{'}(a) \bigr\vert ^{q}+ \frac{1}{4} \bigl\vert f{'}(b) \bigr\vert ^{q} \biggr) ^{\frac{1}{q}} \biggr] . \end{aligned} $$

This completes the proof. □

Setting $\mathtt{k}=1$, $\lambda =\alpha $, $\sigma (0)=1$, and $w=0$ in Theorem 4, we obtain an interesting result, involving the generalized fractional integrals (14) and (15), which is asserted by the following corollary.

### Corollary 6

*Let* $\alpha \in \mathbb{R}^{+}$ *and* $g :[a,b]\to \mathbb{R}$ *be an increasing and positive function on* $[a,b]$ *having a continuous derivative* $g{'}(x)$ *on* $(a,b)$. *Also*, *let* $f :[a,b]\to \mathbb{R}$ *be a differentiable mapping on* $(a,b)$ $(a< b)$ *such that* $\vert f{'}\vert ^{q}$ *is convex for* $q>1$ *with* $\frac{1}{p}+\frac{1}{q}=1$. *Then*

60 $$ \begin{aligned}[b] & \biggl\vert \frac{\Gamma (\alpha +1)}{2} \bigl[ \mathcal{I}_{\frac{a+b}{2} {+};g}^{\alpha }F(b)+\mathcal{I}_{\frac{a+b}{2}{-};g}^{\alpha }F(a) \bigr] -\eta^{\alpha }_{a,b;g} f \biggl( \frac{a+b}{2} \biggr) \biggr\vert \\ & \quad \leq \frac{b-a}{4} \bigl\{ \bigl(\kappa_{p;g}^{\alpha } \bigr)^{\frac{1}{p}} + \bigl(\Lambda_{p;g}^{\alpha } \bigr)^{ \frac{1}{p}} \bigr\} \\ & \qquad {} \times \biggl[ \biggl( \frac{1}{4} \bigl\vert f{'}(a) \bigr\vert ^{q}+\frac{3}{4} \bigl\vert f {'}(b) \bigr\vert ^{q} \biggr) ^{\frac{1}{q}} + \biggl( \frac{3}{4} \bigl\vert f{'}(a) \bigr\vert ^{q}+ \frac{1}{4} \bigl\vert f{'}(b) \bigr\vert ^{q} \biggr) ^{\frac{1}{q}} \biggr] , \end{aligned} $$

*where* $\eta^{\alpha }_{a,b;g}$ *is given in* (57),

$$ \kappa_{p;g}^{\alpha }:= \int_{0}^{1} \biggl[ g(b)-g \biggl( \frac{s}{2}a+ \frac{2-s}{2}b \biggr) \biggr] ^{p\alpha }\,ds $$

*and*

$$ \Lambda_{p;g}^{\alpha }:= \int_{0}^{1} \biggl[ g \biggl( \frac{s}{2}b+ \frac{2-s}{2}a \biggr) -g(a) \biggr] ^{p\alpha }\,ds. $$

### Remark 3

Setting $k=1$ and $g(t)=t$ in Theorem 4 gives the same result as in [21], Corollary 5.

Choosing $k=1$, $g(t)=t$, $\lambda =\alpha $, $\sigma (0)=1$, and $w=0$ in Theorem 4 yields the same result as in [11], Theorem 6.

### Theorem 5

*Let* $\mathtt{k},\rho ,\lambda \in \mathbb{R}^{+}$, $w \in \mathbb{R}_{0}^{+}$, *and* $\sigma (m) \in \mathbb{R}^{+}$ $( m \in \mathbb{N}_{0} ) $ *be a bounded sequence*. *Also*, *let* $g :[a,b]\to \mathbb{R}$ *be an increasing and positive function on* $[a,b]$ *having a continuous derivative* $g{'}(x)$ *on* $(a,b)$. *Further*, *let* $f :[a,b]\to \mathbb{R}$ *be a differentiable mapping on* $(a,b)$ $(a< b)$ *such that* $\vert f{'}\vert ^{q}$ *is convex for* $q\geq 1$. *Then*

61 $$ \begin{aligned}[b] & \biggl\vert \frac{1}{2\mathtt{k}} \bigl[ \mathcal{J}^{\sigma ,\mathtt{k},g} _{\rho ,\lambda ,\frac{a+b}{2}{+};w }F(b) +\mathcal{J}^{\sigma , \mathtt{k},g}_{\rho ,\lambda ,\frac{a+b}{2}{-};w}F(a) \bigr] - \bigl( \varphi _{\rho ,\lambda }^{\sigma ,\mathtt{k},g}(1)+\Phi_{\rho ,\lambda }^{ \sigma ,\mathtt{k},g}(1) \bigr) f \biggl( \frac{a+b}{2} \biggr) \biggr\vert \\ & \quad \leq \frac{b-a}{4} \bigl[ \mathcal{F}_{\rho ,\lambda + \mathtt{k}}^{\sigma_{3},\mathtt{k}} [w]+\mathcal{F}_{\rho ,\lambda + \mathtt{k}}^{\sigma_{4},\mathtt{k}} [w] \bigr] , \end{aligned} $$

*where the notations are given above*:

62 $$ \begin{aligned}[b] \sigma_{3}(m):= {}& \sigma (m) \biggl( \int_{0}^{1}\Delta_{\rho ,\lambda ,m} ^{\sigma ,\mathtt{k},g}(s) \biggr)^{1-\frac{1}{q}} \\ & {}\times \biggl\{ \biggl[ \bigl\vert f{'}(a) \bigr\vert ^{q} \int_{0}^{1}\Delta_{\rho ,\lambda ,m}^{\sigma , \mathtt{k},g}(s) \frac{s}{2}\,ds + \bigl\vert f{'}(b) \bigr\vert ^{q} \int_{0}^{1} \Delta_{\rho ,\lambda ,m}^{\sigma ,\mathtt{k},g}(s) \frac{2-s}{2}\,ds \biggr]^{\frac{1}{q}} \\ & {} + \biggl[ \bigl\vert f{'}(b) \bigr\vert ^{q} \int_{0}^{1}\Delta_{\rho ,\lambda ,m} ^{\sigma ,\mathtt{k},g}(s)\frac{s}{2}\,ds + \bigl\vert f{'}(a) \bigr\vert ^{q} \int_{0}^{1} \Delta_{\rho ,\lambda ,m}^{\sigma ,\mathtt{k},g}(s) \frac{2-s}{2}\,ds \biggr]^{\frac{1}{q}} \biggr\} \end{aligned} $$

*and*

63 $$ \begin{aligned}[b] \sigma_{4}(m):= {}& \sigma (m) \biggl( \int_{0}^{1}\Omega_{\rho ,\lambda ,m} ^{\sigma ,\mathtt{k},g}(s) \biggr)^{1-\frac{1}{q}} \\ & {}\times \biggl\{ \biggl[ \bigl\vert f{'}(a) \bigr\vert ^{q} \int_{0}^{1}\Omega_{\rho ,\lambda ,m}^{\sigma , \mathtt{k},g}(s) \frac{s}{2}\,ds + \bigl\vert f{'}(b) \bigr\vert ^{q} \int_{0}^{1} \Omega_{\rho ,\lambda ,m}^{\sigma ,\mathtt{k},g}(s) \frac{2-s}{2}\,ds \biggr]^{\frac{1}{q}} \\ & {} + \biggl[ \bigl\vert f{'}(b) \bigr\vert ^{q} \int_{0}^{1}\Omega_{\rho ,\lambda ,m} ^{\sigma ,\mathtt{k},g}(s)\frac{s}{2}\,ds + \bigl\vert f{'}(a) \bigr\vert ^{q} \int_{0}^{1} \Omega_{\rho ,\lambda ,m}^{\sigma ,\mathtt{k},g}(s) \frac{2-s}{2}\,ds \biggr]^{\frac{1}{q}} \biggr\} . \end{aligned} $$

### Proof

Using convexity of $\vert f{'}\vert ^{q}$ and the power-mean inequality in Lemma 2, we have

$$ \begin{aligned}[b] \vert \mathcal{L}\vert \leq {}& \frac{b-a}{4} \sum_{m=0}^{\infty }\frac{\sigma (m) w^{m}}{\mathtt{k} \Gamma_{\mathtt{k}}(\rho m \mathtt{k}+\lambda + \mathtt{k})} \\ &{}\times \biggl( \int_{0}^{1} \biggl[ g(b)-g \biggl( \frac{s}{2}a+ \frac{2-s}{2}b \biggr) \biggr] ^{\frac{\lambda }{\mathtt{k}}+\rho m}\,ds \biggr)^{1-\frac{1}{q}} ( \mathcal{R}_{1}+\mathcal{R}_{2} ) \\ & {}+\frac{b-a}{4}\sum_{m=0}^{\infty } \frac{\sigma (m) w^{m}}{ \mathtt{k} \Gamma_{\mathtt{k}}(\rho m \mathtt{k}+\lambda +\mathtt{k})} \\ &{}\times \biggl( \int_{0}^{1} \biggl[ g \biggl( \frac{s}{2}b+ \frac{2-s}{2}a \biggr) -g(a) \biggr] ^{\frac{\lambda }{\mathtt{k}}+\rho m}\,ds \biggr)^{1-\frac{1}{q}} ( \mathcal{R}_{3}+\mathcal{R}_{4} ) , \end{aligned} $$

where

$$\begin{aligned}& \begin{aligned}[b] \mathcal{R}_{1}:= {}& \biggl\{ \bigl\vert f{'}(a) \bigr\vert ^{q} \int_{0}^{1} \biggl[ g(b)-g \biggl( \frac{s}{2}a+ \frac{2-s}{2}b \biggr) \biggr] ^{\frac{\lambda }{\mathtt{k}}+\rho m} \frac{s}{2}\,ds \\ & {} + \bigl\vert f{'}(b) \bigr\vert ^{q} \int_{0}^{1} \biggl[ g(b)-g \biggl( \frac{s}{2}a+ \frac{2-s}{2}b \biggr) \biggr] ^{\frac{\lambda }{\mathtt{k}}+\rho m} \frac{2-s}{2}\,ds \biggr\} ^{\frac{1}{q}}, \end{aligned} \\& \begin{aligned}[b] \mathcal{R}_{2}:= {}& \biggl\{ \bigl\vert f{'}(b) \bigr\vert ^{q} \int_{0}^{1} \biggl[ g(b)-g \biggl( \frac{s}{2}a+ \frac{2-s}{2}b \biggr) \biggr] ^{\frac{\lambda }{\mathtt{k}}+\rho m} \frac{s}{2}\,ds \\ & {} + \bigl\vert f{'}(a) \bigr\vert ^{q} \int_{0}^{1} \biggl[ g(b)-g \biggl( \frac{s}{2}a+ \frac{2-s}{2}b \biggr) \biggr] ^{\frac{\lambda }{\mathtt{k}}+\rho m} \frac{2-s}{2}\,ds \biggr\} ^{\frac{1}{q}}, \end{aligned} \\& \begin{aligned}[b] \mathcal{R}_{3}:= {}& \biggl\{ \bigl\vert f{'}(a) \bigr\vert ^{q} \int_{0}^{1} \biggl[ g \biggl( \frac{s}{2}b+ \frac{2-s}{2}a \biggr) - g(a) \biggr] ^{\frac{\lambda }{\mathtt{k}}+ \rho m} \frac{s}{2}\,ds \\ & {} + \bigl\vert f{'}(b) \bigr\vert ^{q} \int_{0}^{1} \biggl[ g \biggl( \frac{s}{2}b+ \frac{2-s}{2}a \biggr) - g(a) \biggr] ^{\frac{\lambda }{\mathtt{k}}+ \rho m} \frac{2-s}{2}\,ds \biggr\} ^{\frac{1}{q}}, \end{aligned} \\& \begin{aligned}[b] \mathcal{R}_{4}:={} & \biggl\{ \bigl\vert f{'}(b) \bigr\vert ^{q} \int_{0}^{1} \biggl[ g \biggl( \frac{s}{2}b+ \frac{2-s}{2}a \biggr) - g(a) \biggr] ^{\frac{\lambda }{\mathtt{k}}+ \rho m} \frac{s}{2}\,ds \\ & {} + \bigl\vert f{'}(a) \bigr\vert ^{q} \int_{0}^{1} \biggl[ g \biggl( \frac{s}{2}b+ \frac{2-s}{2}a \biggr) - g(a) \biggr] ^{\frac{\lambda }{\mathtt{k}}+ \rho m} \frac{2-s}{2}\,ds \biggr\} ^{\frac{1}{q}}. \end{aligned} \end{aligned}$$

We, therefore, have

$$\begin{aligned} \vert \mathcal{L}\vert \leq &\frac{b-a}{4}\sum _{m=0}^{\infty }\frac{\sigma (m)\vert w\vert ^{m}}{ \mathtt{k} \Gamma_{\mathtt{k}}(\rho m \mathtt{k}+\lambda +\mathtt{k})} \biggl( \int_{0}^{1}\Delta_{\rho ,\lambda ,m}^{\sigma ,\mathtt{k},g}(s) \biggr)^{1-\frac{1}{q}} \\ &{}\times \biggl\{ \biggl[ \bigl\vert f{'}(a) \bigr\vert ^{q} \int_{0}^{1}\Delta_{\rho ,\lambda ,m}^{\sigma ,\mathtt{k},g}(s) \frac{s}{2}\,ds + \bigl\vert f{'}(b) \bigr\vert ^{q} \int_{0} ^{1}\Delta_{\rho ,\lambda ,m}^{\sigma ,\mathtt{k},g}(s) \frac{2-s}{2}\,ds \biggr]^{\frac{1}{q}} \\ &{}+ \biggl[ \bigl\vert f{'}(b) \bigr\vert ^{q} \int_{0}^{1}\Delta_{\rho ,\lambda ,m}^{\sigma ,\mathtt{k},g}(s) \frac{s}{2}\,ds + \bigl\vert f{'}(a) \bigr\vert ^{q} \int_{0}^{1} \Delta_{\rho ,\lambda ,m}^{\sigma ,\mathtt{k},g}(s) \frac{2-s}{2}\,ds \biggr]^{\frac{1}{q}} \biggr\} \\ &{}+\frac{b-a}{4}\sum_{m=0}^{\infty } \frac{\sigma (m)\vert w\vert ^{m}}{ \mathtt{k} \Gamma_{\mathtt{k}}(\rho m \mathtt{k}+\lambda +\mathtt{k})} \biggl( \int_{0}^{1}\Omega_{\rho ,\lambda ,m}^{\sigma ,\mathtt{k},g}(s) \biggr)^{1-\frac{1}{q}} \\ &{}\times \biggl\{ \biggl[ \bigl\vert f{'}(a) \bigr\vert ^{q} \int_{0}^{1}\Omega_{\rho ,\lambda ,m}^{\sigma ,\mathtt{k},g}(s) \frac{s}{2}\,ds + \bigl\vert f{'}(b) \bigr\vert ^{q} \int_{0}^{1} \Omega_{\rho ,\lambda ,m}^{\sigma ,\mathtt{k},g}(s) \frac{2-s}{2} \biggr]^{\frac{1}{q}} \\ &{}+ \biggl[ \bigl\vert f{'}(b) \bigr\vert ^{q} \int_{0}^{1}\Omega_{\rho ,\lambda ,m}^{\sigma , \mathtt{k},g}(s) \frac{s}{2}\,ds + \bigl\vert f{'}(a) \bigr\vert ^{q} \int_{0}^{1} \Omega_{\rho ,\lambda ,m}^{\sigma ,\mathtt{k},g}(s) \frac{2-s}{2}\,ds \biggr]^{\frac{1}{q}} \biggr\} \\ =&\frac{b-a}{4} \bigl[ \mathcal{F}_{\rho ,\lambda +\mathtt{k}}^{\sigma _{3},\mathtt{k}} [w]+ \mathcal{F}_{\rho ,\lambda +\mathtt{k}}^{\sigma _{4},\mathtt{k}} [w] \bigr] . \end{aligned}$$

Finally, we get

$$ \vert \mathcal{L}\vert \leq \frac{b-a}{4} \bigl[ \mathcal{F}_{\rho ,\lambda + \mathtt{k}}^{\sigma_{3},\mathtt{k}} [w]+\mathcal{F}_{\rho ,\lambda + \mathtt{k}}^{\sigma_{4},\mathtt{k}} [w] \bigr] . $$

This completes the proof. □

### Remark 4

Setting $\mathtt{k}=1$, $\lambda =\alpha $, $\sigma (0)=1$, and $w=0$ in Theorem 5 gives a new result, as in Corollaries (5) and (6).

Choosing $\mathtt{k}=1$ and $g(t)=t$ in Theorem 5 yields the same result as in [21], Corollary 7.

Setting $\mathtt{k}=1$, $\lambda =\alpha $, $\sigma (0)=1$ and $w=0$ in Theorem 5 gives the same result as in [11], Theorem 5.
